# Biologically effective dose correlates with linear tumor volume changes after upfront single-fraction stereotactic radiosurgery for vestibular schwannomas

**DOI:** 10.1007/s10143-021-01538-w

**Published:** 2021-04-10

**Authors:** Constantin Tuleasca, Mohamed Faouzi, Philippe Maeder, Raphael Maire, Jonathan Knisely, Marc Levivier

**Affiliations:** 1 Neurosurgery and Neurooncology Service, Centre Hospitalier Regional Universitaire de Lille, Roger Salengro Hospital, Lille, France; 2 Department of Clinical Neurosciences, Neurosurgery Service and Gamma Knife Center, Lausanne University Hospital (CHUV), Lausanne, Switzerland; 3 Faculty of Biology and Medicine (FBM), University of Lausanne (Unil), Lausanne, Switzerland; 4 Signal Processing Laboratory (LTS 5), Ecole Polytechnique Fédérale de Lausanne (EPFL), Lausanne, Switzerland; 5 Division of Biostatistics, Center for Primary Care and Public Health (Unisanté), University of Lausanne, Lausanne, Switzerland; 6 Neuroradiology Department, Lausanne University Hospital (CHUV), Lausanne, Switzerland; 7 ENT Department, Lausanne University Hospital (CHUV), Lausanne, Switzerland; 8 Weill Cornell Medicine, Department of Radiation Oncology, New York-Presbyterian, New York City, NY USA

**Keywords:** Biologically effective dose, Radiosurgery, Gamma Knife, Vestibular schwannoma, Tumor, Volume

## Abstract

Vestibular schwannomas (VSs) are benign, slow-growing tumors. Management options include observation, surgery, and radiation. In this retrospective trial, we aimed at evaluating whether biologically effective dose (BED) plays a role in tumor volume changes after single-fraction first intention stereotactic radiosurgery (SRS) for VS. We compiled a single-institution experience (*n* = 159, Lausanne University Hospital, Switzerland). The indication for SRS was decided after multidisciplinary discussion. Only cases with minimum 3 years follow-up were included. The Koos grading, a reliable method for tumor classification was used. Radiosurgery was performed using Gamma Knife (GK) and a uniform marginal prescription dose of 12 Gy. Mean BED was 66.3 Gy (standard deviation 3.8, range 54.1–73.9). The mean follow-up period was 5.1 years (standard deviation 1.7, range 3–9.2). The primary outcome was changes in 3D volumes after SRS as function of BED and of integral dose received by the VS. Random-effect linear regression model showed that tumor volume significantly and linearly decreased over time with higher BED (*p* < 0.0001). Changes in tumor volume were also significantly associated with age, sex, number of isocenters, gradient index, and Koos grade. However, the effect of BED on tumor volume change was moderated by time after SRS and Koos grade. Lower integral doses received by the VSs were inversely correlated with BED in relationship with tumor volume changes (*p* < 0.0001). Six (3.4%) patients needed further intervention. For patients having uniformly received the same marginal dose prescription, higher BED linearly and significantly correlated with tumor volume changes after SRS for VSs. BED could represent a potential new treatment paradigm for patients with benign tumors, such as VSs, for attaining a desired radiobiological effect. This could further increase the efficacy and decrease the toxicity of SRS not only in benign tumors but also in other SRS indications.

## Introduction

Vestibular schwannomas (VSs) are rising from the Schwann cells, which usually wrap around motor and sensory nerves and, for this particular case, the VIII^−th^ cranial nerve [4]. Patients may experience different specific symptoms, of whom most common is unilateral hearing loss, ringing in the ear (tinnitus), dizziness, facial numbness (if compression of the trigeminal nerve), and rarely facial weakness (if major compression of facial nerve). The apparent increase in incidence has been considered related to increase of earlier diagnosis and incidental finding on magnetic resonance imaging (MRI) [4].

It has been classically considered that VSs are slow-growing tumors, with an average rate of 1 mm/year [23]. However, recent research suggests that the majority of VSs might experience volumetric growth, with about one-third growing at a rate of 100% per year [27]. Management options include observation [22], microsurgical resection [19], or radiation [3, 14]. There is currently no consensus with regard to patient’s selection for therapeutic intervention, although recent studies suggested radiosurgery (in contrast to observation) for small VSs in patients not choosing surgical resection [15]. The outcome goals for both patients and medical staff are tumor control on long-term basis, while preserving cranial nerve function and thus high quality of life.

Single-fraction stereotactic radiosurgery (SRS) is classically recommended for small to medium size VSs [14]. Rates of SRS tumor control are considered similar to those of microsurgical resection, while SRS being less invasive and achieve better preservation of cranial nerves, particularly the facial and cochlear [24]. There was progressive marginal dose prescription de-escalation, to currently 11–13 Gy [26], while attaining equivalent tumor control but with lower morbidity [14, 17]. Recent studies suggested as only predictor for long-term tumor control the initial tumor volume, with 5-year tumor control rate of approximately 91% [13].

It is now well acknowledged that beam on time engaged to deliver same radiation dose may vary significantly. Particularly, for the case of VSs, using multiple isocenters and Gamma Knife (GK), this parameter might vary up by a factor of 10. This aspect is particularly due to decay of the Cobalt-60 sources, with a half-life of 5.26 years. The biologically effective dose (BED), a concept initially used in fractionated radiotherapy, incorporates both the prescribed dose and treatment time [6]. In 1989, Fowler coined the term BED as a linear-quadratic (LQ)–based formula [5]. After 21 years, the same author suggested the wide use of BED in the radiation therapies [6]. Moreover, BED was defined as “the total dose required to give the same log cell kill as the schedule being studied, at an infinitely low-dose rate or with infinitely small fractions well-spaced out, now with an overall time factor for repopulation during continued irradiation. Millar and Canney also proposed this methodology [20, 21]. More recently, application to single-fraction GK was described by Hopewell et al. [9] and further assessed for the outcomes after GK for trigeminal neuralgia (playing a role in pain cessation and complication appearance) [28], for pituitary growth-hormone secreting tumors, the former in the context of decrease in hormone excessive secretion (e.g., in acromegaly, by playing a role in the biological remission) [8] or arteriovenous malformations (by playing a role in obliteration) [29]. However, little is known about the role of BED in tumor volume changes, while prescribing a uniform radiation dose in single-fraction SRS.

Here we investigated, for the first time, whether BED might play a role on long-term 3D tumor volume changes after single-fraction SRS for benign tumors, such as VSs.

## Methods

### Study design and participants

In this retrospective, unicentric study, we analyzed 159 consecutive VSs that have been treated with first intention, single-fraction SRS in Lausanne University Hospital, Switzerland, treated between June 2010 (opening of our GK center) and October 2016.

Tumor control is evaluated on long-term basis after SRS, usually with a minimum follow-up of 2–3 years. In this sense, cases with less than 3 years follow-up were excluded from the present study.

The indication for treatment was always decided after multidisciplinary discussion. Inclusion criteria were as follows: patients aged of at least 18 years old at time of treatment, able to provide written informed consent, treated with first intention SRS for VSs. Were excluded patients with previous microsurgical resection (*n* = 58), pure intracochlear and/or intravestibular (*n* = 4) tumors, cystic VS (reputed to have different radiological answer, *n* = 4) [2], type II neurofibromatosis (*n* = 3), previous radiation (*n* = 1, failed Linac radiosurgery), or a prescribed dose of 11 Gy (*n* = 1, to have uniform dose prescription). Two patients were lost for follow-up. Eighty-five (53.4%) patients underwent single-fraction SRS because of volume increase on serial follow-up MRI, while 74 (46.6%) had either tumors already large at initial presentation (i.e., touching the brainstem) or small but stable tumors with serviceable hearing, assuming that proactive treatment would increase their chances of maintaining functional hearing [25].

Demographic data can be found in Table 1.

**Table 1:** Basic demographic data

Variable	Mean (standard deviation, range)
Follow-up period	5.1 years (1.7, 3–9.2) Minimum 3 years of follow-up: 159 (100%)
*N* patient	159
Age	58.5 years (13.1, 21.1–83.6)
Side	Right: left = 78 (49.1%): 81 (51.9%)
Sex	M: F = 72 (45.3%): 87 (54.7%)
Common presenting symptoms	
• Hypoacusia • Vertigo • Incidental • Tinnitus • Gait problems	• 104 (65.4%) • 20 (12.6%) • 14 (8.8%) • 13 (8.2%) • 8 (5%)
Prior surgery	0 (0%)
Prior irradiation	0 (0%)
Koos grade	
• I • II • III • IV	• 36 (22.6%) • 52 (32.7%) • 68 (42.8%) • 3 (1.9%)
Maximal diameter	14.2 mm (5.5, 4–29.5)
Hearing at baseline (Gardner-Robertson class)	• 1: 71 (44.6%) • 2: 38 (23.9%) • 3: 22 (13.8%) • 4: 5 (3.1%) • 5: 23 (14.5%)

Koos grade [16] was used to characterize the size and anatomical relationship of the VSs. In our practice, we reserve SRS to small to medium size tumors; only 3 patients with large tumors (i.e., Koos grade IV) had SRS, due to contraindication for surgery (*n* = 2) or patient’s choice (*n* = 1). Facial function was normal in all cases at the time of SRS (House-Brackmann grade 1) [10]. The Gardner-Robertson (GR) scale was used to classify hearing function [7]. One hundred and nine (68.5%) patients had useful hearing before SRS (GR class 1 or 2, Table 1). Eighty-nine (81.6%) retained useful hearing at 3 years after SRS.

### Radiosurgery treatment

All patients underwent single-fraction SRS by GK in the modern era of SRS, using completely automatized GK models, including Gamma Knife Perfexion (between June 2010 and until June 2016) and ICON (starting June 2016, Elekta Instruments, AB, Sweden). After frame application under local anesthesia, all underwent multimodal stereotactic imaging, including both MRI (T1-weighted with and without Gadolinium injection (1 mm slices with no gap) and T2 CISS/Fiesta (0.6 mm slices with no gap)) and computer tomography (CT).

A marginal dose of 12 Gy was prescribed in all patients.

The dosimetric data can be seen in Table 2. The mean beam on time was 40.7 min (standard deviation (std dev) 17.7, range 7.3–101.8). The mean tumor volume was 1.1 cc (std dev 1.4, range 0.01–7.8). The mean integral dose received by the tumor volume was 18.7 mJ (std dev 23.2, range 0.2–116.6).

**Table 2:** Basic dosimetric data at the time of radiosurgery

Variable	Mean (standard deviation, range)
Dose	12 Gy (100% of patients)
BED	66.3 Gy_2.47_ (3.8, 54.1–73.9)
Beam on time	40.7 min (17.7, 7.3–101.8)
Number of isocenter	9.7 (7.2, 1–32)
Radiation dose rate	2.7 Gy/min (0.6, 1.7–3.8)
Tumor volume	1.3 mL (1.5, 0.03–8.5)
Integral dose received by the tumor volume	18.7 mJ (23.2, range 0.2–116.6)
Maximal dose received by the cochlea	4.3 Gy (1.5, 1.5–10.5)
Gradient index	3 (0.5, 2.4–5.8)

There was no treatment interruption during the GK single-fraction sessions.

### Primary aim

The primary outcome was changes in 3D volumes after SRS as function of BED.

### Biologically effective dose computation

The mean BED was 66.3 Gy (std dev 3.8, range 54.1–73.9). The BED was computed using the basic BED model, which takes into account the beam on time and the prescribed dose. Of note, all patients received the uniform marginal dose of 12 Gy.

Biologically effective dose values were calculated using similar approaches, as proposed by Jones and Hopewell [12]. We have initially applied such calculations to monoisocentric plans (trigeminal neuralgia) [28]. We further suggested that BED can be a better predictor as compared with the prescribed marginal dose for obliteration of arteriovenous malformations after single-fraction radiosurgery, using a uniform dose prescription [29]. Recently, we also evaluated the BED’s role in endocrine remission, in the context of GK for acromegaly [1].

### Biologically effective dose analysis function of primary outcome

The BED was analyzed as a continuous variable in relationship with 3D volume changes (please see below). A secondary analysis was conducted using BED as by third quartiles.

Moreover, BED was analyzed as a continuous variable for the probability of progression at 3 years after SRS (a change in volume between therapeutic moment and 3 years after SRS of more than 0%, binary outcome—stable or decrease versus increased) but also for the probability of further surgical intervention.

### Follow-up period and protocol

The mean follow-up period was 5.1 years (std dev 1.7, range 3–9.2). Fifty-three (33.3%) of cases had 5 years follow-up MRIs and 51 (32.1%) had 7 or 9 years follow-up MRIs.

The neurosurgery team (CT, ML) performed regular follow-up at 6, 12, 36, 60, 84, and 108 months, respectively. Each patient underwent brain MRI and, if pre-SRS useful hearing, audiologic assessment. All patients had been seen in person. The same scales as defined in the “Study design and participants” section were used during follow-up period.

Radiosurgery failure was defined as the need for additional treatment, with second SRS, microsurgery, or a combination of both techniques. The main indication for such approach was tumor progression noted during consecutive measurements and potential symptomatic mass effect. The final decision whether to retreat a patient or not was made in multidisciplinary discussion.

### Volumetric analysis

All volumes (3D) were measured within the Leksell Gamma Plan (LGP, Elekta Instruments, AB, Sweden) using a standardized procedure, by manual contouring, by the same neurosurgeons (ML, CT). The T1-weighted Gadolinium injected (1 mm slice) image during follow-up was available for each patient and was co-registered with the therapeutic one. The automated segmentation tool from the LGP was used to contour the VS on each slice, slice by slice, and corrected manually when appropriate (e.g., segmentation including non-VS contrast-enhancing structures, such as dura and vessels).

**Fig. 1:**
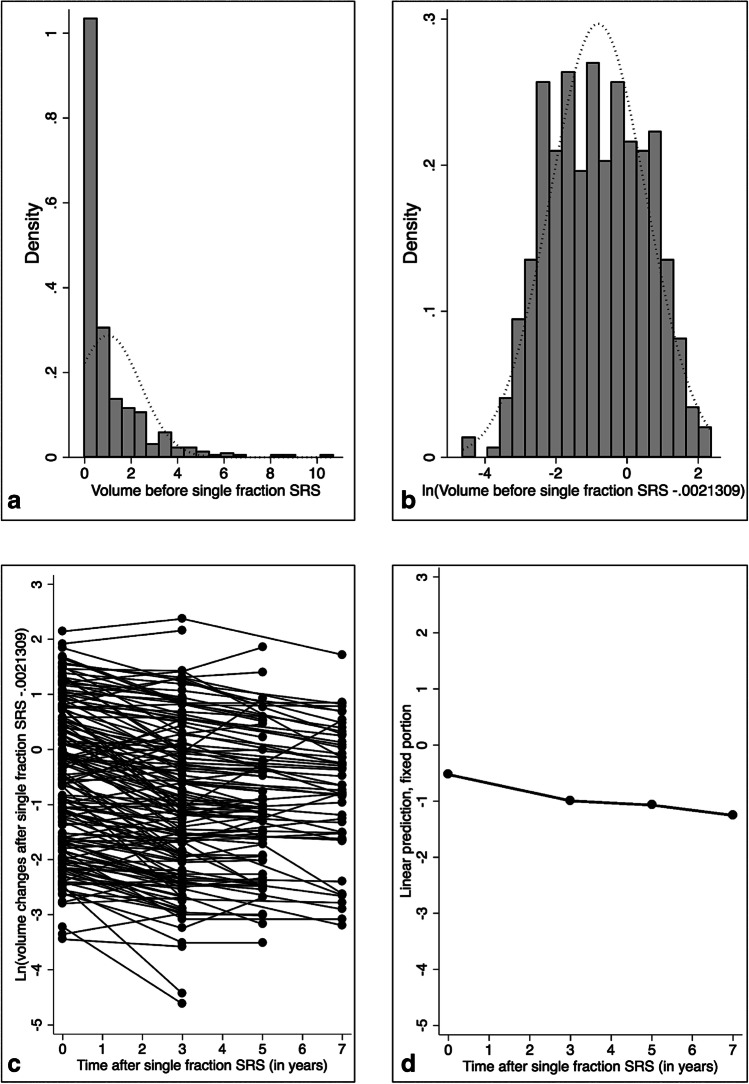
The therapeutic tumor volumes were not normally distributed (Fig. 1a); in this respect, a logarithmic transformation was done (Fig. 1b ); the mean tumor volume at the time of SRS receiving the radiation dose and during follow-up course (**c** for each individual patient and **d** for all patients, censored at 7 years)

The volume was noted individually and at each time point, in milliliters (Fig. 1a).

### Statistical analysis

Statistical analysis was performed using Stata 14 (StataCorp, College 109 Station, Texas). Descriptive statistics were related as proportion/frequency for categorical data and mean, median, and range for continuous variables. The therapeutic tumor volumes were log transformed to get a normal distributed outcome (Fig. 1b). Random-effect linear model was then used to assess the association between the outcome, the time point after SRS (time, 3, 5, 7, or 9 years), the BED, and covariates of interest.

First, univariable associations were tested for each covariate as well as their potential interactions.

Then, significant predictors at a 20% level were considered in a backward procedure selection to fit a multivariable model. Let ${V}_{ij}$ the tumor volume of the subject $i$ measured at ${time}_{ij}$ and ${X}_{i}$ be a covariate (like *age*, *sex*,…) or interaction between two covariates (like *BED* × *Koos grade* or *BED* × *time*) of subject $i$ measured at Gamma Knife intervention. The fitted random-effect linear model was:

$${V}_{ij}=\left({\beta }_{0}+{u}_{i}\right)+ {\beta }_{1 }{Time}_{ij}+ {\beta }_{2 }{BED}_{i }+{\beta }_{x }{X}_{i }+{\varepsilon }_{ij}$$

where ${\beta }_{0}$ is the global mean, ${\beta }_{1}$, ${\beta }_{2}$, and ${\beta }_{x}$ are the fixed population parameters that measure respectively the effect of time, *BED* and covariate $X$ on the tumor volume. The ${u}_{i}$ is the individual-specific random effect and ${\varepsilon }_{ij}$ the independent measurement errors with mean zero.

To illustrate the dynamics of tumor volume decrease after SRS according to the BED value, we additionally coded the BED in three categories (1st, 2nd, 3rd quartiles) and plotted the linear prediction by BED quartile over time.

## Results

### BED and tumor volume decrease

The mean tumor volume at the time of SRS was 1.3 mL (std dev 1.5, range 0.03–8.5) (Table 3, Fig. 1, c—for each individual patients at baseline and with the further variations during follow-up), at 3 years was 0.9 mL (std dev 1.3, range 0.01–10.7), at 5 years 0.7 mL (std dev 1.1, range 0.03–6.4), and at more or equal to 7 years was 0.8 (std dev 0.9, range 0.04–5.6, Fig. 1, d—for all patients, censored at 7 years).

**Table 3:** Vestibular schwannoma volumetric evolution after radiosurgery and the need for further intervention

Variable	Mean (standard deviation, range)
Tumor volume at the time of single-fraction radiosurgery	1.3 mL (1.5, 0.03–8.5)
Volume at 3 years after single-fraction radiosurgery (*n* = 159, 100%)	0.9 mL (1.3, 0.01–10.7)
Volume at 5 years after single-fraction radiosurgery (*n* = 53, 33.3%)	0.7 mL (1.1, 0.03–6.4)
Volume at > = 7 years after single-fraction radiosurgery (*n* = 51, 32.1%)	0.8 mL (0.9, 0.04–5.6)
Further intervention (surgery or single-fraction radiosurgery)	6/159 (3.4%)

**Fig. 2:**
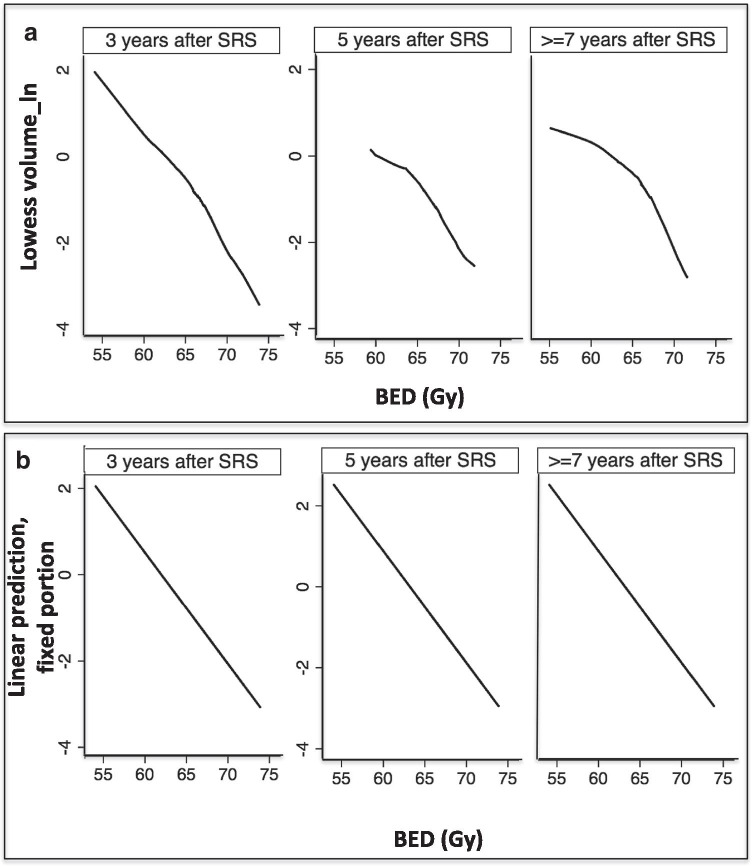
Volume decrease during follow-up course at 3, 5, and 7 years after single-fraction SRS and function of BED

The univariate analysis showed that increasing the BED by one unit was associated with tumor volume decrease (log-scaled) of − 0.27 (95% confidence interval (CI) [− 0.31, − 0.24], *p* < 0.0001) in average (Fig. 2). Moreover, tumor volume decreased by a mean of − 0.47 (95% CI [− 0.55, − 0.38], *p* < 0.0001), − 0.55 (95% CI [− 0.68, − 0.41], *p* < 0.0001), and − 0.72 (95% CI [− 0.87, − 0.59], *p* < 0.0001) respectively at 3 years, 5 years, and 7 years follow-up. Other covariates associated with tumor volume that decrease after SRS were age, sex, Koos grade at the time of SRS, radiation dose rate, and the number of isocenters (Table 4).

**Table 4:** Uni- and multivariate analysis

Variable	Univariate analysis			Multivariate analysis		
	Coefficient Beta (Odd’s ratio)		*p*-value	Coefficient Beta (Odd’s ratio)		*p*-value
Changes in volume (as continuous variable) after single-fraction radiosurgery						
Age		− 0.013 (0.98)	0.09	− 0.008 (0.992)	0.007	
Sex (male = 0)		− 0.016 (0.98)	0.9	0.24 (1.27)	0.004	
Koos grade (I + II as reference)						
• III + IV		3.17 (23.8)	< 0.0001	-6.90 (0.001)	< 0.0001 < 0.0001 0.002	
Time						
• 3 years • 5 years • > = 7 years		− 0.49 (0.61) − 0.54 (0.58) − 0.72 (0.48)	< 0.0001 < 0.0001 < 0.0001	− 1.99 (0.13) − 3.09 (0.04) − 4.24 (0.01)	< 0.0001 < 0.0001 < 0.0001	
BED		− 0.27 (0.76)	< 0.0001	− 0.17 (0.84)	< 0.0001	
Integral dose received by the tumor volume		0.044 (1.04)	< 0.0001			
Radiation dose rate		0.43 (1.53)	0.008			
Number of isocenters		0.14 (1.15)	< 0.0001	0.053 (1.05)	< 0.0001	
Gradient index		− 1.77 (0.17)	< 0.0001	− 0.67 (0.51)	< 0.0001	
BED × Koos grade		–	–	0.11 (1.11)	0.001	
BED × Time		–	–	0.008 (1.008)	< 0.0001	

After adjustment of significant covariates, multivariate model showed that tumor volume decreased in average by − 0.17 (95% CI [− 0.23, − 0.11], *p* < 0.0001) per BED unit (Table 4). Moreover, the time point after SRS and the Koos grade at the time of treatment moderated the observed 3D volumetric change (Table 4).

**Fig. 3:**
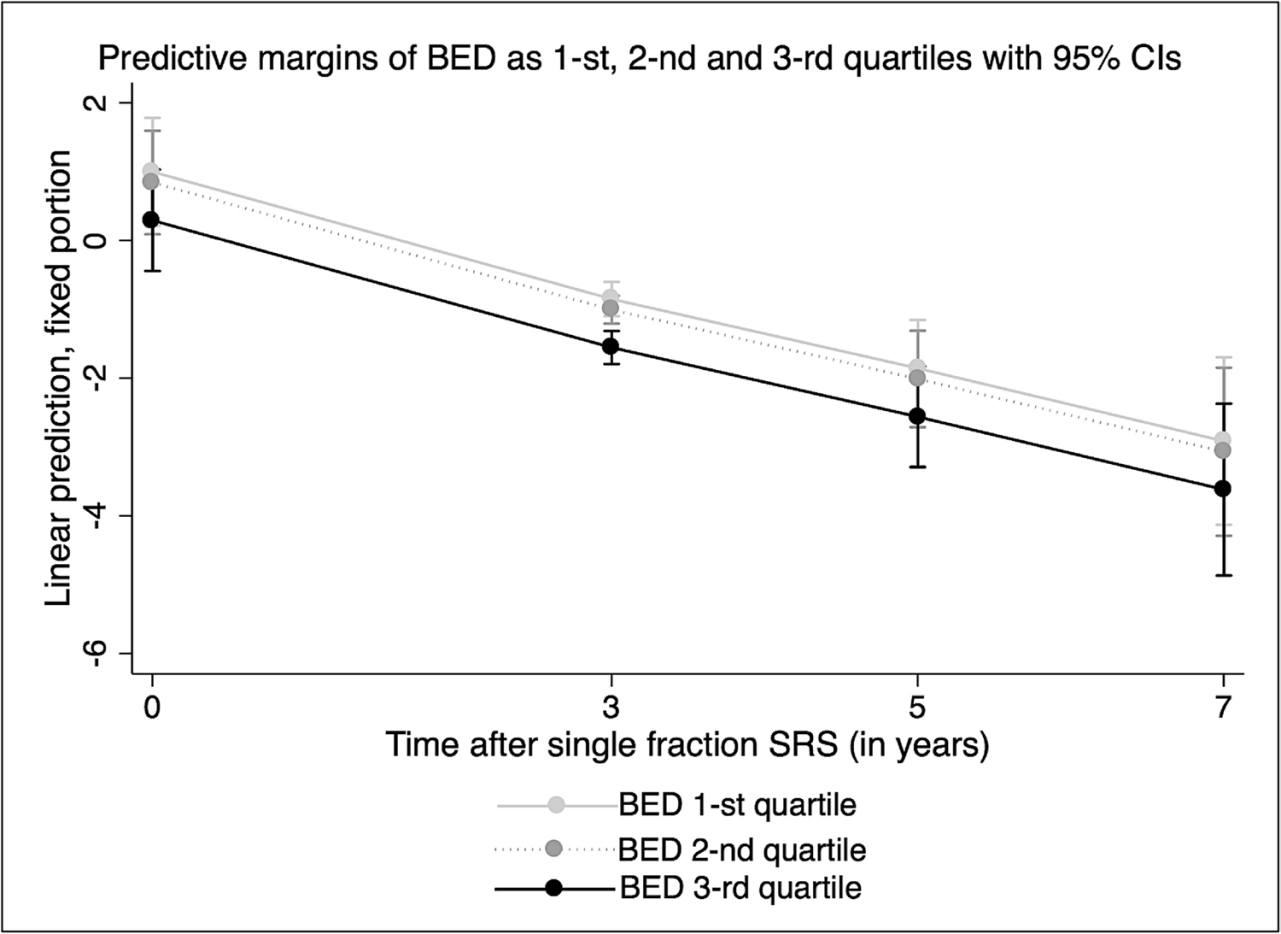
Volumetric decrease during follow-up course at 3, 5, and 7 years after single-fraction SRS is more important for BED in 1st, 2nd, and 3rd quartiles

The dynamics of tumor volume decrease after SRS according to the BED value by three categories 1st, 2nd, and 3rd quartiles can be seen in Fig. 3 (*p* < 0.0001).

### BED and further intervention

Six (3.4%) patients needed further intervention, of whom repeat SRS was considered in 2 and planned subtotal microsurgical resection followed by SRS in 4. There was no statistically significant relationship between the probability of further intervention and BED (Table 3, *p* = 0.54), although the limited sample size precluded a multivariate analysis.

### Integral dose received by the tumor volume

**Fig. 4:**
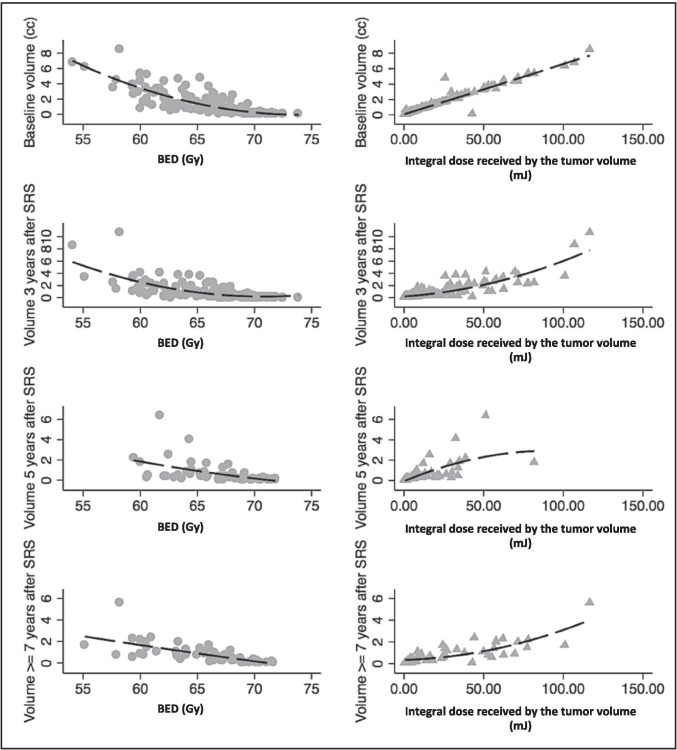
From left to right, correlations between VSs’ volumes (from up to down at baseline, 3, 5, and >  = 7 years) and BED (left), integral dose received by the tumor volume (right) (*p* < 0.0001)

The integral doses received by the tumor volume were inversely correlated with BED. Moreover, oppositely to BED, higher integral doses received by the tumor volume were associated with VSs increase after SRS (*p* < 0.0001, Fig. 4).

## Discussion

This retrospective analysis met its primary endpoint and showed that BED (ranging between 54.1 and 73.9 Gy) is related to volumetric 3D changes after single-fraction, first intention SRS for VSs, treated with a uniform radiation dose of 12 Gy, for a beam on time varying between 7.3 and 101.8 min. The overall volumetric changes are supported by the durability of responses during a long-term follow-up period and can, therefore, be considered long-term control. Biologically effective dose was statistically significant both as a continuous value, but also evaluated as 1st, 2nd, and 3rd quartiles as related to volumetric changes after SRS. The integral dose received by the tumor volume was inversely correlated with BED with regard to volumetric changes.

The BED is a relatively modern concept, usually accounting for the effects of fractionated radiotherapy, taking into account the different behaviors of early- and late-reacting tissues. In many clinical contexts, radiobiological considerations can be usefully applied. It has been initially defined as the total dose required to give the same log cell as the schedule being studied, at an infinitely low-dose rate or with infinitely small fractions well-spaced out, taking into account the overall time factor for repopulation during continued irradiation [6]. Thus, clinicians should be aware about developing such a quantitative radiobiological approach in their practice [11]. In classical radiotherapy, specific attention should be given to whether the dose per fraction is being altered, possible “hot spots” within the planning, combination of treatments, different histological classes for which is required a different alpha/beta ratio [11]. Moreover, there is a wide expanding use of the BED concept in brachytherapy, radiochemotherapy, dose escalation studies, radionuclide-targeted therapy, or for quantifying any treatments using ionizing radiation [6].

Here and to the best of our knowledge, we used BED to evaluate volumetric tumor changes after single-fraction SRS for benign tumors, such as VSs. It is now well acknowledged that beam on time will become progressively longer due to the decay of Co-60 sources and their half-life of 5.26 years, when using GK for SRS. Moreover, even for a fixed source activity, the same effect would be attained by plugging/blocking beam channels, to protect vital structures, such as the cochlea (for hearing preservation in the case of VSs treated by SRS) [18], or the optic apparatus (to avoid post-SRS optic neuropathy). Additional aspects such as collimator factors or inter-patient-specific geometry might further influence treatment time, hence the need of the BED, incorporating both delivered dose and the beam on time.

One of the main strengths of the present study is the size of the treated cohort (more than 100 patients), treated in a uniform manner, in a single center. Moreover, all patients have been treated with SRS devices that work in an automated manner, with no need to account for additional time, such as collimator changes, as this could have been the case for former GK models (i.e., B, C, etc.). The follow-up period of at least 3 years and the durability of the radiological response are further key points of such analysis. Also, the uniformity of measurements and their slice by slice contouring make the reported volumes reliable and reproducible.

The limitations of the present study are related to the inherent one pertaining to the retrospective cohorts. Another aspect is the use of a simplified BED calculation, using a mono-exponential fit. Yet, our purpose was to provide the reader with a clinically relevant analysis with a translational impact. A third limitation is that our findings are pertinent only to single-fraction first intention SRS. In this sense, previously irradiated or operated VSs might have a specific and different radiobiological profile.

## Conclusions

Overall, the encouraging results obtained here suggest a significant relationship between BED and tumor volume changes after single-fraction SRS. This could represent a potential new treatment paradigm, while aiming at a desired radiobiological effect and prescribing a certain BED value for an individual patient, as modulating the prescribed dose by the factor time. Further studies should validate such an approach on larger cohorts.

## Data Availability

N/A.

## Code availability

N/A.
